# Moving goals. Goal-work in Parkinson's disease rehabilitation

**DOI:** 10.3389/fresc.2022.819862

**Published:** 2022-08-10

**Authors:** Merete Tonnesen, Claus Vinther Nielsen, Rikke Sand Andersen

**Affiliations:** ^1^Department of Public Health, Aarhus University, Aarhus, Denmark; ^2^DEFACTUM, Central Danish Region, Aarhus, Denmark; ^3^Department of Clinical Social Medicine and Rehabilitation, Region Hospital West Jutland, Herning, Denmark; ^4^Department of Anthropology, Aarhus University, Aarhus, Denmark; ^5^Department of Public Health, University of Southern Denmark, Odense, Denmark

**Keywords:** goal-work, Parkinson's disease, rehabilitation, chronic disease, goal-setting

## Abstract

Chronic diseases often demand considerable work by patients: they must adhere to medical regimes and engage with social and embodied discontinuities. In Denmark, rehabilitees in Parkinson's disease rehabilitation talk about Parkinson's as their new job. In this article, we introduce *goal-work* as an optical lens to enlarge and explore the micro-social practices that concern a core practice in rehabilitation where professionals and rehabilitees set goals for the future and work toward the goals. To work with goals adds a new task to living with Parkinson's. Rehabilitation research tends to focus on the actual goal-setting meeting. Drawing on data from long-term ethnographic fieldwork on goals and their setting in Parkinson's disease rehabilitation, we show how participants in rehabilitation imagine, set, enact, review or share their rehabilitation goals, and how goals are worked with before and after the goal-setting meeting, across settings. We conceptualize these micro-social practices as goal-work, which we argue is a spatio-temporal process. The concept of goal-work emphasizes the fact that goal-setting is one event in a string of goal-related activities, and it turns our attention to the intersubjective dimensions inherent in goal-work, such as the role of relatives and how acts of imagination and acts of sharing form part of goal-work.

## Introduction

In a rehabilitation context, a goal can be defined as a future state to be achieved through rehabilitation activities (1, 2). As the future is not yet, imagination becomes a vessel to access the future; participants in goal-setting tour the future in order to determine which goals they should aim for (3). Goal-setting may be viewed as a temporal practice with an imaginative component to it.

A time-gap exists between the present, when goals are set, and the future, when they may be achieved. This means that when goals are set in the present, it opens a space of potentiality that can be worked with. However, as we will show, this space of potentiality can also evaporate; goals may not always actualize. The effort to achieve rehabilitation goals has many moments of uncertainty attached to it. Yet this is the work task for participants in rehabilitation.

In rehabilitation, goal-setting serves multiple purposes, e.g., establishing a direction of the rehabilitation trajectory, making transparent the link between components in the process, enhancing patient autonomy, enabling change to be monitored, and demonstrating adherence to e.g., professional requirements (1, 4). As has been described in the literature, phases in the goal process include preparation, negotiation, goal-setting, goal pursuit, and review (5, 6). Goal-related activities thus “span the whole continuum of service delivery from admission, to implementation of treatment, to evaluation of progress and outcome” [(7), p. 12]. However, researchers have pointed to a gap in the literature. A scoping review on goal-setting among chronically ill individuals found that 39 of 58 included articles did not pay attention to the phases prior to and after the actual goal-setting (8). Yet in order to fully comprehend the phenomenon of working with rehabilitation goals there is a need to describe and analyze the continuum of goal related activities, thus adding contemporary observational data to studies by e.g., Struhkamp (9) or Mattingly (3).

Another gap in the goal literature concerns Parkinson's disease (PD), though studies have been published on the actual goals set in PD rehabilitation (10–12). Considering that neurological disorders are “the leading source of disability in the world, and PD is the fastest growing of these disorders” (13), it appears relevant to add ethnographic studies to literature on goal-setting in PD rehabilitation.

PD is a progressive, neuro-degenerative disease which has no cure. PD symptoms include a range of motor and non-motor symptoms, rigidity, tremor, problems with cognition, speech, depression, sleeping disorder, and apathy (14, 15). The symptoms progress. PD can be called a designer-disease, tailored each individual (16). An experience of embodied uncertainty enshrouds life with PD (17); fluctuations happen over time and even within the day, which makes it difficult to trust one's body (18).

Chronic illness entails chronic work. Living with PD entails work such as exercise, adjusting medicine, getting out of bed when low on medicine and dealing with a body that one cannot rely on (16). As the disease progresses and the body changes, social relationships also change with spouses taking over tasks and caring; managing PD can be “a collective undertaking” [(18), p. 59], as also found in Warren and Sakellarious' writing about motor neurone disease and PD and the intersubjectivities of care (19). Intersubjective means that individuals are never isolated, but related to others, to structures and contexts (20).

The progressive character of PD makes it difficult to match the etymological meaning of the *re* in rehabilitation, i.e., “to come back to.” Though functioning may improve, PD rehabilitation often concerns maintaining functioning for as long as possible. PD demands continuous work, adapting to the progression of the disease. Goal-work adds to this on-going work. The term goal-work is inspired by informants expressing how, “Parkinson's disease is my new job,” “training is a job,” and anthropological studies linking chronic illness and work, e.g., the “chronic home-work” that takes place in people's lives when illness becomes part of life (21) and the “work of care” that relatives do in caring for and caring about (22). Extending this line of thinking about care, Andersen et al. (23) note how informal caring is not a one-way stream, but requires sharing.

The aim of this article is two-fold. The primary aim is to introduce the concept of goal-work as a way to conceptualize the different and manifold micro-social practices that goes into the goal process in a Danish rehabilitation center and beyond, including the phases before and after the formal and institutionalized goal-setting meeting. Another aim is to add knowledge about goals and their setting in PD rehabilitation, i.e., a situation with declining functioning. To our knowledge, this article is the first to describe and analyze how goals are worked with in PD rehabilitation.

Goal-work refers to the multiple micro-social practices that rehabilitees, their relatives, and professionals engage in as they set, pursue, review, and share goals, using different skills and methods. In this article, we conceive of work as an intentional activity that requires effort and attention, be it social, sensory or cognitive. *Work* stimulates an attention to the who (workers), the where (workplaces), and the how (tasks involved, skills required). We emphasize that goal-work is a spatio-temporal process, meaning that goals are worked with in different settings and over time, and relating to the fact that goals are often imagined and set in the present for the future, yet drawing on experiences from the past. Our conceptualization of goal-work focuses on micro-social activities and also point to the intersubjective dimensions of goal-work, in the sense that goal-work requires acts of sharing intimate and private insights (23).

## Methodology

This ethnographic study was designed as a multi-sited fieldwork, as coined by Marcus (24). Fieldwork took place from January 2019-December 2020, and the first author worked with a total of 20 key-informants and their social networks. This included being “hospitalized” with them and follow them and their goals from a rehabilitation unit to their homes, local physiotherapist clinics and neurologists at hospitals and private clinics.

### Sampling methods and setting and informants

The first author entered the field through a Danish unit for specialized rehabilitation (Sano), being “hospitalized” with two different groups of persons with Parkinson's Disease, 20 persons in all, throughout their rehabilitation stay. Rehabilitees' ranged in age from their 50s to 70s. Besides PD, some rehabilitees had heart disease, osteoporosis, two were cancer survivors, and several had rheumatic diseases. The 20 persons lived in different regions of Denmark; all but three lived with spouses. They had diverse occupational backgrounds; four were still working, three of them part-time.

**Figure 1:**
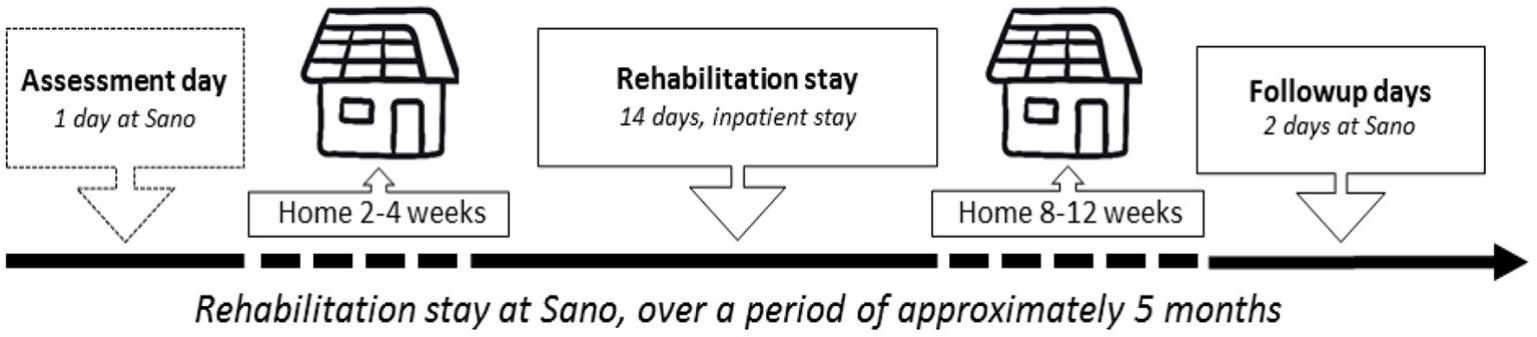
A rehabilitation process including periods at the rehabilitation center and at home.

Approximately 12.000 persons live with Parkinson's disease in Denmark, with an average age of diagnosis of 60–62 years. Denmark has free health and social services, financed by general taxes. PD rehabilitation at Sano is thus free, eligible for persons (with no dementia) in phase two “the maintenance phase,” where symptoms increase, typically gait problems and phase three, “the complex phase” with e.g., fluctuations and hyperkinesia, depression, problems regarding self-care, dysphagia, decreased mobility, and hallucinations [(25), pp. 43, 44]. PD rehabilitation at Sano takes place over several months, starting with an assessment day, followed a few weeks later by a two-week in-patient stay with a goal-setting meeting on the first day; after a couple of months, there is a 2-day follow up stay (Figure 1). The rehabilitation course combines group sessions (training/educational) with individual sessions with a physiotherapist (PT), an occupational therapist (OT), and a nurse. The following tests are repeated three times: timed up and go, 5 times sit to stand, and a 6-min walk test. The inter-professional staff members are required to work with rehabilitation goals, as Sano adhere to the Danish White Paper on rehabilitation (4), and to the professional guidelines that emphasize goal-setting as part of the rehabilitation process, e.g., European Physiotherapy Guideline for Parkinson's disease (26). They use no particular goal-setting tool [as e.g., Goal Attainment Scale as described by Kiresuk and Sherman (27)].

In a multi-sited fieldwork, field-sites often materialize during fieldwork. In order to follow the flow of goals over settings, rehabilitees were asked to map their present PD landscape, i.e., the situations and places of importance when living with PD. All 20 rehabilitees participated in this. The mapping pointed to home as an essential setting, together with neurological clinics and physiotherapy clinics. Neurologists are the medical specialists central to PD trajectories (general practitioners refer PD related matters to the neurologist) with consultations once or twice a year (a few less, a few more), about 30 min per consultation. The majority of rehabilitees attend group-based physiotherapy once a week. These were thus settings where goals might be worked with. *Via* the rehabilitees, a purposeful sampling of neurologists and physiotherapists that rehabilitees in this study consulted was made. The plan was to explore if they worked with goals or showed interest in rehabilitees' goals. Contacting them to ask for interviews coincided with the Covid-19 pandemic, which impacted their work. Some did not return calls, some declined due to lack of time, or lock-down restrictions.

### Methods

#### Participant-observation

A study of goals in rehabilitation requires an attention to spoken, enacted, and written goals. At the Sano rehabilitation center entailed participating in training sessions (morning gym, gym, Nordic walking, boxing, voice training), attending educational sessions (on PD, on training, etc.), and observing individual sessions between a rehabilitee and professional staff member(s). To live, to do and to be together, provided an insight to living with Parkinson's, and helped us crystallize our concept of goal-work. To reverse the view from rehabilitees to professionals, three inter-professional teams (each with nurse, physiotherapist, occupational therapist) were followed on goal-setting days, and several staff meetings were attended. Participant observation outside Sano involved attending local training sessions with physiotherapists, Parkinson's dances and observation during consultations with neurologists. Participant observation in 2019–2021 includes approximately 2 months at Sano, following two full courses. Observations include 90 individual consultations, 85 at Sano and 5 at neurologist consultations and physiotherapy group sessions.

#### Interviews

In total, 64 semi-structured interviews and a substantial number of unstructured interviews were conducted with 20 rehabilitees, 6 relatives and 29 professionals attached to the Sano course and beyond. All these interviews were structured by an interview guide and audio-taped. Two semi-structured interviews were conducted with each of the 20 rehabilitees, first during their hospitalization at Sano, and again approximately 2 months later in their home, before the follow-up stay; here the spouses sometimes participated in part of the interview. Rehabilitees were asked about their illness narratives to provide an understanding of their rehabilitation stay from a wider perspective, with reflections on their lives before and after diagnosis, and about their experience of setting and working with goals. They were asked to map their PD landscape, sometimes *via* drawings on a piece of paper. Spouses were asked about living with PD as a relative, and about their thoughts on PD rehabilitation goals. Eighteen Sano professionals were interviewed *via* focus group interviews and six professionals (two had also participated in focus-group interviews) participated in in-depth interviews. An additional seven external neurologists and physiotherapists have participated in in-depth interviews.

Several unstructured interviews were conducted with most rehabilitees, either contacting them with follow-up questions, or to hear how they were doing, or they phoned to have a chat. Over time, multimodal data such as text messages, e-mails, paintings, photos and poems entered the study from rehabilitees or from their relatives. Notes were taken in all unstructured interviews.

#### Document analysis

An analysis of rehabilitees' records was conducted to describe demographic and health data of rehabilitees, to compare the written documentation with observational data on goal-work, and finally to make an analysis of documented goals using ICF as a way to classify goals. In this article, we primarily draw on data from observations and interviews.

### Analysis

Data were analyzed through an iterative process that involved reading the research literature, revisiting research questions, and analyzing data from observations and interviews (28). Notes from informal conversations, observation, and unstructured interviews and text messages and e-mails have been coded together with transcribed interviews. A thematic analysis was made, both to find some common denominators in the data, for instance “work” and “sharing” that appeared as themes across informant groups and settings but also being attentive to specific themes that may shed light on nuances of goal-work, though not necessarily mentioned by the majority of informants. Data were also analyzed chronologically, i.e., notes and transcriptions from each rehabilitee from assessment day to months after the end of the course were analyzed and compared with data from other rehabilitees' trajectories. Patient journals were also consulted in the analysis. The chronological analysis was made to track how goals were formulated and altered over time, to distinguish goal phases in a trajectory and to analyze across rehabilitee trajectories the modus operandi during each activity, e.g., assessment days, scheduled goal-setting meeting, in talks with e.g., a nurse regarding goals, or consultations with neurologists. Coupling chronological and thematic analysis enabled an understanding of the flow of goals over time and space and paved way to pick the scenes that best illustrate the findings.

### Ethics

The American Anthropological Association' principles of ethical obligations for anthropological work guided the project. A verbal introduction to this study was supplemented with a written description, including ethical matters, such as access to own transcripts and procedure to exit the study if so wished. Participants provided written consent to use data from interviews, observations, and patient records in an anonymized form. Fieldwork included access to intimate details of persons' lives, shared in confidentiality. In order to ensure anonymity, all names described in the examples below are pseudonyms and specific goals or identifiable traits of a person have been slightly changed.

## Findings

### Goal-work

**Figure 2:**
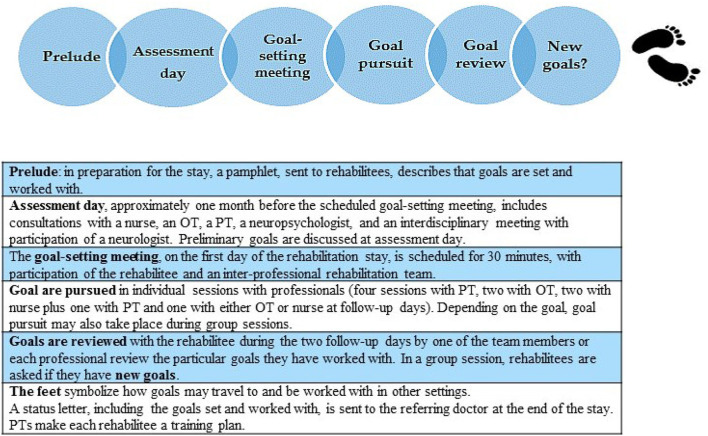
Goal process at Sano rehabilitation center.

In the following, we illustrate goal-work through particular scenes from fieldwork. We attend closely to how the goal process unfolds in an ordinary everyday rehabilitation context in order to elucidate the outreach of rehabilitation goals and the multiplicity of doings surrounding rehabilitation goals. The scenes are chosen among 90 individual consultations observed because they represent noteworthy aspects of what goal-work can entail. They will be recognizable across different rehabilitation settings, yet rarely analyzed in detail in contemporary goal-literature. Furthermore, the scenes illustrate how differently PD shows and affects everyday life. Some find PD devastating to their everyday life within a few years after diagnosis, while others experience a slower progression. The continuous progression of the disease makes it difficult to distinguish whether symptoms relate to PD, aging, or to the side-effects of medication. A great deal of work goes into training the body, the voice, and skills, trying to postpone a future that will entail a degeneration of functioning. Incentives for this kind of work emanate from a fear of becoming a vegetable, as many informants said, someone who cannot move nor participate in social life. To obtain a sense of the flow of goals in a rehabilitation trajectory, we present the scenes in the same chronological order that informants would normally experience, (see Figure 2 below).

Analysis across goal-setting meetings exposes a common modus operando. As hosts, professionals set the scene and open up the meeting, the person who writes down the goals on the PC often facilitates the meeting, inviting the rehabilitee to present goals. Some come prepared with goals, others do not, in which case the team mentions the goals discussed at assessment day. Professionals emphasize that they try to use a person's own words in the documented goals.

The 20 rehabilitees, our informants, had lived with PD for some years before attending the rehabilitation program. When asked why they applied for the program, they responded 1) to get a boost, mentally and especially physically, 2) to meet other people with PD, and 3) to gain knowledge about the disease.

### David: Scenes from assessment day

*During the assessment day, the person with PD, often accompanied by a relative, meet an inter-professional team. Preliminary goals are discussed*.

David, a man in his 70s, has brought his wife Anette to the assessment day. He was diagnosed with Parkinson's 3 years ago, but believes his symptoms appeared 10 years prior to that. In the consultations during the day, he meticulously responds to professionals' questions and explains how his changing body affects everyday life. He finds his cognition impaired, has problems with his diaphragm, he experiences pain, has a bent-over posture, he easily falls, has difficulty turning around in bed at night, and his walking and dancing abilities have weakened. Asked about freezing episodes, a sudden inability to move, he says, “I see others worse off than me.” Anette adds to the picture: “Remember to mention your hip problems […] since the diagnosis you find it hard to go for walks. […] You also have problems with your vision—double-vision.”

After consultations with an OT, a PT, a nurse, and a neuro-psychologist, the final meeting of the day is an inter-professional consultation where a neurologist also participate. Upon leaving the room, the OT asks David to wait:

OT: I have made a print-out of some of the goals we have talked about. I will go and fetch it for you.
David: Are they your goals or my goals?
OT [smiling]: Those are just preliminary goals based on our talk. Then we have a point of departure for the first day of your rehabilitation stay. They are changeable—take a look at them at home.

During assessment days, each talk or examination add new layers of information as professionals try to obtain a picture of who the rehabilitee is and how he or she manages everyday life. Sometimes spouses contribute with information, like Anette, exposing a “shared doctoring” (29), as participants work together to add parts to the bigger picture in order to find the appropriate interventions, and thus exposing the intersubjective dimension to goal-work.

In the rehabilitation process, the assessment is followed by goal-setting (30). In this scene, goals materialize as topics of conversation during the assessment. In the words of the OT, the goals are “preliminary” and “changeable.” David's long-term goal is to keep dancing, short-term goals concerns maintaining ability to go for walks and improve balance to enable participation in dance lessons. Although David nodded in assent during the summary discussion, when the preliminary goals were mentioned he was still skeptical: whose goals are they? This could suggest an insecurity as to the purpose of goals and for whose sake they are set; or it could exemplify what David plainly states as “I don't always get what is going on.” Generally, preliminary goals are mentioned, maybe printed and handed over to the person with PD, with a word of encouragement to do “homework” and think about goals. In reflections about assessment day, a number of rehabilitees found that their goals were actually set that day, while others did not remember talking about goals (though fieldnotes showed that goals were mentioned).

### Peter: The goal-setting meeting

*Goals are imagined, discussed, set (or consolidated), negotiated, and documented at goal-setting meetings. In patient journals, under the heading “treatment plan,” goals are listed together with a list of mainly group sessions which can be ticked off. Most of the 20 rehabilitees set three short-term goals, about half set a long-term goal*.

Peter, in his 60s, has lived with Parkinson's for more than a decade. He lives with his wife. In his part-time job, he is used to set and work with organizational goals. He comes well prepared for the goal-setting meeting with his team. “I have my goals here”, he says, pointing to his head.

Nurse: Do you remember we spoke about you writing some sentences that contain the goals you'd like to work with? [Peter nods in agreement]. We wrote some things down at the assessment day, but maybe you've had some new ideas?
Peter: Yes, but let's take what you wrote as a starting point.
They discuss the goals. Regarding the first goal, “walk more smoothly to be able to carry a plate 15 meters,” Peter explains that he trips (almost stumbles) a lot, “and if only you can help me with the tripping—that would solve the rest.” The PT asks what less tripping would enable him to do, which opens for a glimpse of Peter's imagining: going shopping and going for walks with his wife in a forest nearby their home. The second goal, “minimize sleep interruptions', is agreed upon immediately and without any discussion. Regarding the third goal, “to get up and down a chair without help”, Peter stands up in order to illustrate his problems rising from a chair or the toilet. They discuss his balance and after a while, the OT says: “Did you bring some goals?” “Yes Incontinence.” They discuss what the problem is and the nurse explains that there are several means to handle incontinence. The fourth goal becomes “making it to the toilet in time.”
Finally, the nurse points to the computer and says that she has written everything down. Peter replies: “Okay, as long as you don't show it to anyone.” Pointing at her colleagues and me, the nurse says affirmatively, “we have all signed a confidentiality agreement”. “As long as my employer does not know this” “No, this is your paper”. The goals are printed and handed to Peter.

Peter's case illustrates several aspects common to goal-setting meetings observed. Firstly, how imagination is part of goal-work. Goal-setting requires acts of imagination, i.e., participants have to imagine scenarios for the future. To direct activities to match Peter's goals, the PT had to gain access to what Peter imagined being able to do if his tripping lessened (going for walks with his wife). Secondly, how sharing of information (e.g., imaginings of the future, everyday losses encountered and intimate bodily changes) is inherent to goal-setting. Bladder dysfunction, a common problem in PD (15), is an embarrassing issue for Peter, which he only shared with the team because they might be able to help. Information may be shared beyond the persons participating in the meeting. The PC symbolizes a connective capacity to other settings and to other people. It is not unusual that rehabilitees question the further flow of written words, i.e., with whom the electronic correspondence connects. In some instances, the computer is transformed into a dangerous vessel that can distribute information about a person's most intimate issues. This touches on ethical aspects of goal-work. Thirdly, setting goals seems to be a fluid process: goals are discussed at assessment day and documented as preliminary goals in patient journals. Some rehabilitees do homework, making a list of goals at home, new goals may appear during the goal-setting meeting, and goals discussed on the assessment day are reiterated and refined. There is thus a build-up phase to goals. Finally, goals are manifested in writing, handed over manually as a print out to rehabilitees, which can both be interpreted as empowering, as handing over ownership of goals, or as a pragmatic reminder of the goals agreed on, or it can signify a contract between the team members (including the rehabilitee). Several rehabilitees referred to goals as a contract made with themselves and with their team. It should be noted, that while Peter came prepared and readily set goals, others found goal-setting less straightforward for different reasons; unacquaintance with goal-setting and its purpose, problems with imagining goals for a future that seems uncertain regarding how fast will PD progress, or lack of knowledge about viable goals, e.g., which goals could be worked with at Sano.

### Morten: Goal-pursuit. Nurse consultation during the two-week rehabilitation stay, scheduled as a “talk about coping”

*In individual consultations, rehabilitees and professionals pursue goals. At times, other matters of importance materialize*.

Morten in his late 60s, was diagnosed with PD 4 years previously. In their first session, the nurse asks, “Have you thought of something you'd like to talk about? Otherwise I have, but I'd like to hear from you first.” Morten would like to work with physical activity—“that's the problem, I lost motivation.” His training has almost come to a halt. One of his goals is to get a physical boost, with a long-term goal stated as “To get motivated to keep up my training and regain belief that what I do matters.” Morten and the nurse gradually unravel what made him lose motivation: an operation and how “for the past 5 years, things have gone downhill, I have to fight to not let things go further downhill.” His words indicate how, with neuro-degenerative diseases, goals may be set to *maintain* rather than *improve* functioning.

Morten: I get depressive spells at times. On some days, my body simply won't cooperate. Some days I put on my clothes [to do training], but I find my body to be too stiff, and I get angry.
[It takes effort to keep a body with PD agile, the nurse agrees, probing into Morten's anger and discussing strategies for the “rainy days,” when Morten gets depressed]. Morten: I just have to get out of the house, with the mood I have, and keep on going. I know *you* have an understanding, but people who don't have PD, they don't know what it feels like. Yesterday at a session, we heard about all the things that are wrong with us—when relatives notice this, they want us to change it. My wife gets irritated with me—you know when I leave things, and she feels she has to clean up my stuff.
Nurse: Can you talk about this?
Morten: Some days, yes. I understand why she gets annoyed.
Nurse: That's tough on both of you, but each in your own ways. You live with it, and your wife observes it, that can be tough.
Morten: I used to be the patriarch of the family, but no more [His voice trembles, tears roll down his cheeks]. And she would like me to be. I haven't stopped playing this role. I try, but things are not the way they used to be.

Asked about his family's reactions to his diagnosis, Morten cries, “It's not easy to talk about … there is a lot of going downhill. My problem is I'm so sad that I cannot be me.” The nurse gets up from her chair, places a hand on Morten's shoulder, and says some soothing words. They have gone beyond their scheduled time. The nurse suggests that Morten shares his thoughts with his family.

This case illuminates several aspects of goal-work. One concerns how affect and managing one's emotions form part of micro-social activities of goal-work. Morten shows emotions as he laments his identity loss, sharing how he feels forced to recast his identity. The nurse embraces the emotions, having a catalog of strategies at her disposal (listening, calm voice, smile, eye contact etcetera), exposing how goal-work is embodied. Another concerns the navigational skills involved in finding what matters to the individual and how an intimate space created between a rehabilitee and a professional can bring to light issues of importance that may not have materialized during the goal-setting meeting. Relatedly, goals may follow a linear line, such as one of Morten's short term goals “to get a physical boost and be able to do 1,5 km walks in the nearby park again, on a regular basis.” This goal was set, worked toward with the PT, evaluated, and achieved, even if 1,5 km became 1 km. Other goals are less linear and a goal-talk can take new directions. In this case, Morten's goal about motivation and what to do when the motivation fails open the talk, but then other issues of importance come to the forefront and the conversation narrows down to what really matters to Morten, i.e., his loss of identity, a common theme in interviews with informants, and also described elsewhere (17, 31, 32). Finally, this case exemplifies how the past (being a family patriarch), present (“cannot be me”) and future (going downhill) entangle.

### David: Goal-pursuit and review at home, between rehabilitation stay and follow-up days

*Goals travel over time and may be set, pursued or reviewed at settings beyond the rehabilitation setting, e.g., at home*.

On David's goal-sheet, his long-term goal is to “keep participating in the PD dance,” whereas short-term goals are to “be able to walk with less pain, and continue to walk without any aids, except for Nordic walking sticks for longer walks” and “improve balance to enable participation in dance lessons,” resembling the goals discussed during the assessment day. During his stay at Sano, he told me: “I can't imagine a nice life with a rollator, I just can't. Of course that's where it ends, but somehow I hope I die before that […] the things I've written down bother me on a daily basis. And if these could be eliminated, I might experience a better quality of life.” However, he also expressed some concerns, he had expected his team to take his hand and lead him toward achieving his goals, but feels unsure whether “the tools they provide me with here are that helpful—but time will tell.” Two months later, in an interview at David's home, I ask him to tell if his goals matter to him.

David: Probably yes, but… it might matter more to Sano than to me. That sounds harsh. And I'm not quite sure about it… I've never been good at setting goals. So setting goals like that was new to me. I wrote it all down before going to Sano.
Anette (his wife who left work early to join the interview): We discussed [your goals] when we came from assessment day, and then you added onto the goals before leaving for the 14-day course.
*And during those 14 days—how did you all work toward your goals?*
David: I think what the nurse gave me [suggestions for pain control] was most useful. And someone suggested that I change physiotherapist if I was not happy with my local group-based training.
Anette: If I can add to your thoughts, the goals were to continue going for walks and dancing. And I think both of us find that there was not enough focus on those goals.
At the end of the interview, David reflects on his rehabilitation stay: “I had expected more, I think… but as time passes, I notice that I did get something out of it after all.” He mentions pain management (and shows a medication schedule), exercises (and points to the floor in the living-room: “I've bought a yoga mat and that big ball. Those exercises relieve my pain”). He now does his Parkinson's training at a new physiotherapist (who is “much better”), and uses breathing exercises given by the physiotherapist at Sano (that “really helps”).

The scene exemplifies the spatio-temporal aspect of goal-work, i.e., how goals travel from rehabilitation setting to home as some rehabilitees take their goals home with them and continue working on them. In this scene, the goals took on physical manifestations: a yoga mat and a medication schedule, both technologies used to work toward achieving the goals. It also shows how spouses may be co-workers, doing joint home-work by discussing possible goals before the stay, reviewing goals and assessing the goal-work performed by professionals at Sano. This illustrates the intersubjective dimension to goal-work. In this case, the couple had expected more focus on specific goals, which may indicate a more general observation during fieldwork that steps taken in the goal-pursuit may seem obvious to professionals, yet appear less visible to rehabilitees. In another case, a husband incited to go on strike, as he believed his wife's exercises to achieve her goals were too tiring.

### Bodil. Goal-pursuit and review with OT at follow-up days

*Goals are pursued and reviewed in individual consultations*.

The last scenes are with Bodil, a woman in her mid-70s. She lives on her own, and was diagnosed with PD 4 years ago. Her long-term goal is to be able to balance between daily tasks at home and training so she has energy to pursue her hobbies. Her short-term goals are to improve walking and “organize and plan everyday tasks *via* a weekly schedule.” We meet at an apartment at Sano, where Bodil changes bedlinen, an increasingly demanding task, in pursuit of this particular goal. The OT explains they will repeat the test from last time, asking how Bodil has been doing since her stay.

Bodil: It's been a bit messy, but I've really tried [working with goals].
The OT asks detailed questions: where does she keep the bedding—in a drawer, right, so put it in a drawer here, etcetera, trying to imitate a home situation. Bodil fights her way against the linen, while the OT records her observation on a piece of paper. ‘I don't get this, what's wrong', Bodil whispers to herself, while struggling with the linen. Finally, finishing with the top sheet, she says, ‘I could have joined the military!' We smile. ‘Oh, I did it in the wrong order.' She had forgotten to put the duvet cover on. She seems more and more stressed.
OT: I see that at times you become doubtful—does that happen at home?
Bodil: This is a new situation.
OT: How do you think it went?
Bodil: Fine, considering it's a new place, sometimes I get a bit confused if many things happen at the same time.

We could say that the scene exemplifies the cognitive impairment that is common in PD, but it would perhaps be more accurate to call it a loss of embodied knowledge; the loss of being able to perform those taken-for-granted activities that we carry out without thinking about them. Making the bed is an embodied life task that Bodil has done for years without thinking about it. Now, this and other seemingly easy tasks have become difficult. The scene generates both an understanding of what we could call “detective work”: “Does that happen at home?,” the OT asks, in order to establish the extent of the problem, and also why professionals at Sano underscore the importance of observation. They often experience a difference between what people say and do.

The scene also shows how goal-review can occur in a very subtle way. The OT assesses the goal achievement *via* the test and by asking Bodil how she worked with the goal at home. However, Bodil's PT reviewed her goal-list, evaluating with Bodil whether goals were achieved and discussing a plan for the future, which was a contact with her local municipality to continue rehabilitation. This shows the spatial aspect to goal-work, linking to new professionals in other settings to “take over” and linking the bed linen situation at Sano to the one at home. Based on observation, goal-review seemed in some cases almost invisible. Exploring this finding in interviews with professionals, they pondered on their practice. They agreed that goal-review during follow-up days may not always appear visible to rehabilitees, as it was done in consultations with a professional, not with the team, nor was it scheduled as part of the program.

### Bodil's first consultation at the neurologist after rehabilitation stay

*Goals may travel to other settings and be shared—or they may not*.

We wait with in the hospital corridor. A sign says 'Neurological Unit.' A colorful painting contrasts the white walls. It is called 'Hunting for Dopamine.' Persons with PD lack dopamine. Bodil evaluates her stay at Sano—she was happy to go, but she found it difficult to implement the advice given. Once seated in the neurologist's office on each side of a desk, Bodil tells the neurologist she has been to Sano. The neurologist asks a few questions about her stay, but none about her goals. The two then review the list of questions that Bodil has prepared.

Rehabilitation goals travel from Sano with the rehabilitee as a medium, or through a discharge letter sent from Sano *via* electronic communication to the doctor who made the referral to Sano. Discharge letters encompass goals set, a resume of interventions pursued with each professional and strategies suggested. Judging from this and other observations and interviews with rehabilitees and neurologists, neurologists took no particular interest in rehabilitation goals. One neurologist said: “I do read the discharge letter from Sano, but I think the goals tend to be similar, like walking?” This was correct: the majority of goals among informants in this study were activity and participation related, with a large majority concerning the ability to walk or move around. Goals related to bodily functions mostly concerned sleep and cognitive challenges. A temporal aspect may also influence an interest in goals—in Bodil's case, her appointment with the neurologist took place 4 months after her stay at Sano, her PD symptoms had deteriorated, and she had a long list of questions.

This scene exemplifies how and where goals travel or move forward and the engagement (or lack of engagement) in rehabilitation goals among various specialists. Following goals beyond the inpatient rehabilitation center showed how goals that stretched beyond the stay were mainly “hand-carried” by the rehabilitee to other settings. Many, like Bodil, presented their training plan from Sano to their local physiotherapist, who then incorporated Sano suggestions into their training program.

## Concluding discussion

Through the representation of scenes from the Danish Parkinson's Disease Rehabilitation context, we have shown that the goal process entails different temporal, social and spatial dimensions, which may be conceptualized as goal-work. The scenes expose how PD can shake taken-for-granted assumptions about life—what used to be ordinary embodied knowledge and routines have become exhausting, difficult tasks. Several issues of living with PD impact rehabilitation and thus goal-work, e.g., good days and bad days, apathy, cognitive impairment, depressive spells, and also a need to keep active.

By attending closely to practice, to the phases prior to, during and after the formalized goal-setting meeting, we exposed some of the micro-social activities involved and the inter-subjective and spatio-temporal dimensions of these activities. Levack et al. (33) call goal-setting a complex intervention, and the scenes described display how all the micro-activities of goal-work require a diverse variety of skills and tools, not just for professionals, but for all involved, including communicational and observational skills. We have shown that goals move across settings and that goal-work changes character during the rehabilitation process. Workplaces included the rehabilitation center, the home environment and the local physiotherapist clinic. Some rehabilitees did home-work to prepare for or work with goals, thus adding to the chronic home-work involved in living with PD (21). Goals moved, aided by an infrastructure such as the electronic documentation system. We showed how rehabilitees, professionals and spouses enacted goal-work in speech acts, in writing, and in mundane everyday tasks such as making a bed or during more profound emotional processes involving the family.

Goal-work entailed shared work, as participants worked together trying to find a way, setting and working toward the goals in a rehabilitation trajectory with many possible roads to pursue. Finding the right way was not always a straightforward task. While professionals at Sano are steadfast that the goals should be the rehabilitee's, rehabilitees at times found it difficult to figure out what professionals wanted when they asked about goals, which interventions were offered at Sano, indeed, whose goals were formulated and discussed—the professionals or the rehabilitees. Shared work entails shared responsibilities, professionals and some rehabilitees underscored, comparing their goals with having a contract. The invocation of *contract* may indicate a moral imperative to work toward the goals, with the expectations not only from professionals, but also from a personal expectation toward one's own work.

We have shown that although the goal process has a certain linearity to it, the process of setting goals is fluid, as goals are not necessarily set at the scheduled goal-setting meeting, but maybe at assessment day, at home with the spouse, or after the goal-setting meeting, suggesting a quasi-linear goal-process. In one of the scenes, an intimate space was created by a nurse and a rehabilitee which paved way for talking about sensitive issues, not revealed during the goal-setting meeting. This shows how goals may only appear in intimate rooms but also how a goal-talk can form an entry into other issues of importance. We have shown how goals are materialized or enacted, and how they travel, i.e., move forward, and also how they may come to a dead end.

Furthermore, part of the goal process, review of goals, happened in a subtle way. Professionals reviewed goals, but while goal-setting was scheduled and interdisciplinary, reviews were un-scheduled and uni-disciplinary, and in some cases hardly noticeable. In several interviews, rehabilitees expressed doubt that their goals had been reviewed. Nevertheless, they all believed that once their goals had been set, that they should be subject to evaluation. This underscores the importance of a certain calibration of expectation and more transparency in the steps being taken, as these steps may be unclear or invisible to rehabilitees (and spouses). So which insights does this study offer the rehabilitation field concerned with goals? We will stress particular insights that concern micro-social activities such as acts of imagination and sharing, and the embodied and intersubjective dimensions to goal-work.

We could call goal-setting a technology of imagination, because to set goals requires a view to an imagined future. As proposed by Mattingly (3), the practical actions necessitate an orientation from imagined endings. Professionals rely on rehabilitees to share their imagination in order to support the steps toward the goals. Thinking about goals as imagined lends way to understand that while goals represent a space of potentiality, goals do not necessarily follow a linear line, from set to pursued to achieved. Things happen, the imagined may not materialize and goals may be discarded or forgotten.

Acts of sharing among participants also form part of goal-work; sharing concerns tasks, responsibilities and information about the everyday losses encountered, worries, or intimate issues. Goal-work is thus embedded in sociality as Andersen et al. (23) note, from studies of cancer patients, how information sharing is enmeshed in “social risks and notions of selfhood,” and those who receive care must commit to involving “oneself in difficult and sometimes emotional situations” [(23), pp. 2,13]. Rehabilitees may share tasks, information and uncertainty (34) with spouses and professionals—or they may not. As depicted in the scenes, sharing involved a delicate and ethical balancing. For staff, this balancing concerned which information to share across sectors, how to ask questions in a respectful way, refraining from overstepping an invisible discretion line, or how to handle rehabilitee goals that seem out of sync with reality. For rehabilitees, balancing implied deciding which information to share. Acts of sharing can involve a risk once documented, because where does information flow and who might gain access to the information? While goal-work seems to require acts of sharing, it should therefore be remembered that to decide what to share is hardly straightforward or self-evident.

Goal-work is embodied, intersubjective, and reflected individual life circumstances. It involves senses, emotions, cognition, and is enacted through the body in the practical activities. It takes emotional skills for professionals to do goal-work.

Families can be part of goal-work. This insight is not new, but it is an under-researched theme in the goal literature (35). In our study, some spouses were co-workers in goal-work. They added information to the assessment, helped in the homework process by discussing goals, collaborated in setting and reviewing goals, or they reminded their spouses to do goal-work. Home-visits revealed that the participation spousal co-working was common, making goal-work “a collective undertaking” with the family involved (18). While the role of family in goal-work differs from one PD rehabilitee to another, it nevertheless remains a factor which rehabilitation professionals must acknowledge.

### Strengths and limitations

A strength of our study is that it is based on a long-term ethnographic fieldwork which allowed an exploration of goal-work over time and settings, using a range of different methods. Following 20 persons over two years provided rich data concerning goal-work and living with PD. We also find it a strength that we represent an ordinary practice of working with goals, rather than testing a “polished” model. Working with goals is complex, with no recipe of *the* right way of doing it. However, paying attention to the ordinary may expose obvious flaws in a practice, for instance as described a need to make goal-review explicit to all involved. Describing this may inspire others to reflect on their practice.

There are some limitations. Covid-19 did, to a certain extent, affect the exploration of how goals were worked with after Sano. Though we used information from rehabilitees to support the data obtained from neurologists and physiotherapists, it would have enriched data if the original plans for fieldwork could be followed. Furthermore, even though a rehabilitation stay is free of charge, it has to be applied for, which may result in socially skewed access. We do not claim that our sample of 20 informants is representative of people in rehabilitation, but the richness of fieldwork data, in terms of the number of goal-setting meetings, social interactions and situations observed during 2 years of intense data generation, ensures study validity. We have chosen to depict a few carefully selected scenes from the many situations and interactions observed. In order to represent the temporal and spatial aspects of goal work, we introduce a few informants of the 20 key-informants; each case is illustrative of general issues which arose from our analysis. We aimed to introduce goal-work as a way to conceptualize the manifold micro-social practices in a goal process, and to add knowledge about goals and their setting in PD rehabilitation. While we believe that the concept of goal-work may be applicable in a wide range of rehabilitation settings, as a useful framework to open up and discuss components of the goal-process, the particular findings concerning PD may not be directly applicable to other settings, even if the findings will probably find resonance among professionals working with persons with degenerative diseases. While the scenes illustrate aspects of goal-work, the activities of goal-work are not exhausted in our cases, nor do we pay much attention to organizational structures, discourses, regulations, logics, or obstructions that might influence goal-work, all of which are matters of importance in the PD rehabilitation process.

In conclusion, we found the concept of goal-work a valuable framework for analyzing the work of goals as a process, with goal-setting as an event in that process. Goal-work allows for an attention to the different workplaces, the broad variety of micro-social activities involved and the persons involved, exposing the spatio-temporal and intersubjective aspects of goal-work. The title of our article is “Moving Goals.” Moving indicates how goals are dynamic, on the move, aided by infrastructure, and moved by participants over time and settings. Moving also refers to the emotions so clearly moved by goal-work, as persons with PD imagine and orientate to an uncertain future.

### Clinical implications

There are some clinical implications of our findings: Goal-work may take place in settings beyond the actual rehabilitation setting and be a collective undertaking with spouses or other professionals taking active part. To coordinate and support goal-work, clinicians could uncover who is involved, and if considered appropriate and approved by the rehabilitee, involve co-workers more openly. As the rehabilitation and goal-work processes may be unclear to some rehabilitees (and spouses), clinicians must share their expectations of responsibility in the process and make explicit and visible the how and why of actions, including the connection between goal and intervention. Inter-professional teams must review goals with the rehabilitee (what have we done, why and how did it go), clearly dividing tasks between them (who does the review and how). Clinicians make use of emotional and ethical skills in their work. Rehabilitation management should acknowledge and support this type of work, incorporating these themes in staff-meetings or staff-education.

Further research is needed into the dynamics of how goals move between settings, how clinicians across settings share goals, and how participants in rehabilitation divide goal tasks between them.

## Data availability statement

The datasets presented in this article are not readily available because informants are guaranteed full anonymity.

## Ethics statement

Ethical review and approval was not required for the study on human participants in accordance with the local legislation and institutional requirements. The patients/participants provided their written informed consent to participate in this study.

## Author contributions

MT is the primary author, the article is based on her fieldwork. CVN is the second author and RSA is the last author. Data analysis was done by MT, development of the argument and the analytical concept, and goal-work was done in collaboration between all three authors. MT and RSA did most of the editing of the article. All authors contributed to the article and approved the submitted version.

## Funding

This study was funded by a full Ph.D. scholarship from Department of Public Health, Aarhus University, Denmark (6424).

## Conflict of interest

The authors declare that the research was conducted in the absence of any commercial or financial relationships that could be construed as a potential conflict of interest.

## Publisher's note

All claims expressed in this article are solely those of the authors and do not necessarily represent those of their affiliated organizations, or those of the publisher, the editors and the reviewers. Any product that may be evaluated in this article, or claim that may be made by its manufacturer, is not guaranteed or endorsed by the publisher.

## References

[1] Siegert RJ Levack W. Rehabilitation Goal Setting: Theory, Practice, and Evidence. Boca Raton, FL: CRC Press, Taylor and Francis Group (2015).

[2] Wade DT. Evidence relating to goal planning in rehabilitation. Clin Rehabil. (1998) 12:273–5. 10.1191/026921598678166365 9744662

[3] Mattingly C. Healing Dramas and Clinical Plots: The Narrative Structure of Experience. Cambridge, MA: Cambridge University Press. (1998).

[4] Marselisborgcentret. *Rehabilitering i Danmark: Hvidbog om rehabiliteringsbegrebet [Rehabilitation in* Denmark: White Paper on Rehabilitation]. Marselisborgcentret (2004).

[5] Rauch A Scheel-Sailer A. Applying the international classification of functioning, disability and health to rehabilitation goal setting. In: Siegert RJ Levack W editors. Rehabilitation Goal Setting: Theory, Practice and Evidence. p. 161–180). Boca Raton, FL: CRC Press, Taylor and Francis Group (2015).

[6] Rosewilliam SPA Roskell C. Goal setting for stroke rehabilitation. In: Siegert RJ Levack W editors. Rehabilitation Goal Setting: Theory, Practice, and Evidence. Boca Raton, FL: CRC Press, Taylor and Francis Group (2015).

[7] Levack Siegert. Challenges in theory, practice and evidence. In Siegert RJ Levack W. (editors). Rehabilitation Goal Setting: Theory, Practice, and Evidence. Boca Raton, FL: CRC Press, Taylor and Francis Group. (2015).

[8] Lenzen SA Daniëls R Van Bokhoven MA Van Der Weijden T Beurskens A. Disentangling self-management goal setting and action planning: a scoping review. PLoS ONE. (2017) 12:e0188822. 10.1371/journal.pone.0188822 29176800 PMC5703565

[9] Struhkamp R. Goals in their setting a normative analysis of goal setting in physical rehabilitation. Health Care Anal. (2004) 12:131–55. 10.1023/B:HCAN.0000041187.93819.17 15487815

[10] Kang E Jethani P Foster ER. Person-centered goal setting is feasible in people with parkinson's disease who have subjective cognitive decline: a mixed methods study. Disabil Rehabil. (2022) 13:1–8. 10.1080/09638288.2022.2025930 35023794 PMC9719695

[11] Vlagsma TT Koerts J Fasotti L Tucha O van Laar T Dijkstra H. Parkinson's patients' executive profile and goals they set for improvement: Why is cognitive rehabilitation not common practice? Neuropsychol Rehabil. (2016) 26:216–35. 10.1080/09602011.2015.1013138 25693688

[12] Watermeyer TJ Hindle JV Roberts J Lawrence CL Martyr A Lloyd-Williams H. Goal setting for cognitive rehabilitation in mild to moderate parkinson's disease dementia and dementia with lewy bodies. Parkinson's Dis. (2016) 2016:8285041. 10.1155/2016/8285041 27446628 PMC4942668

[13] Collaborators GPD. Global, regional, and national burden of Parkinson's disease, 1990-2016: a systematic analysis for the Global Burden of Disease Study 2016. Lancet Neurol. (2018) 17:939–53. 10.1016/S1474-4422(18)30295-3 30287051 PMC6191528

[14] Kudlicka A Hindle JV Spencer LE. Everyday functioning of people with parkinson's disease and impairments in executive function: a qualitative investigation. Disabil Rehabil. (2018) 40:2351–63. 10.1080/09638288.2017.1334240 28597694

[15] Obeso JA Stamelou M Goetz CG Poewe W Lang AE Weintraub D. Past, present, and future of parkinson's disease: a special essay on the 200th anniversary of the shaking palsy. Mov Disord. (2017) 32:1264–310. 10.1002/mds.27115 28887905 PMC5685546

[16] Solimeo S. (2009). With Shaking Hands: Aging with Parkinson's Disease in America's Heartland. New Brunswick, NJ: Rutgers University Press.

[17] Warren N Ayton D. (Re)negotiating normal every day: phenomenological uncertainty in Parkinson's disease. In: Sakellariou D Thomas GM editors. Disability, Normalcy, and the Everyday (1 edition). New York, NY: Routledge. (2018). p. 142–57.

[18] Nijhof G. Uncertainty and lack of trust with Parkinson's disease. Eur J Public Health. (1996) 6:58–63. 10.1093/eurpub/6.1.58

[19] Warren N Sakellariou D. Neurodegeneration and the intersubjectivities of care. Med Anthropol. (2020) 39:1–15. 10.1080/01459740.2019.1570189 30707041

[20] Mattingly C. Critical phenomenology and mental health: moral experience under extraordinary conditions. Ethos. (2019) 47:115–25. 10.1111/etho.12230

[21] Mattingly C Grøn L Meinert L. Chronic homework in emerging borderlands of healthcare. Cult Med Psychiatry. (2011) 35:347–75. 10.1007/s11013-011-9225-z 21695552

[22] Manderson L Warren N. “Caring for” and “Caring About”: Embedded interdependence and quality of life. In: Manderson L Warren N editors. Reframing Disability and Quality of Life: A Global Perspective. Amsterdam: Springer (2013). p. 179–93.

[23] Andersen RS McArtney J Rasmussen BH Bernhardson BM Hajdarevic S. Caring as Sharing. Negotiating the Moral Boundaries of Receiving Care. Critical Public Health. (2020) 30:567–76. 10.1080/09581596.2019.1623381

[24] Marcus GE. Ethnography in/of the world system: the emergence of multi-sited ethnography. Annu Rev Anthropol. (1995) 24:95–117. 10.1146/annurev.an.24.100195.000523

[25] DanModis. PARKINSONS SYGDOM. Klinisk Vejledning. Diagnose, forløb og behandling fra et tværfagligt perspektiv. Denmark: DanModis (2011).

[26] Keus S Munneke M Graziano M Paltamaa J Pelosin E Domingos J. European Guidelines for Physiotherapy in Parkinson's Disease. ParkinsonNet/KNGF (2013).

[27] Kiresuk TJ Sherman RE. Goal attainment scaling: a general method for evaluating comprehensive community mental health programs. Community Ment Health J. (1968) 4:443–53. 10.1007/BF01530764 24185570

[28] Hammersley M Atkinson P. Ethnography: Principles in Practice. London: Routledge (2007).

[29] Mol A. *The Logic of* Care: *Health and the Problem of Patient Choice*. London; New York, NY: Routledge. (2008).

[30] Wade DT. Describing Rehabilitation Interventions. Clin Rehabil. (2005) 19:811–8. 10.1191/0269215505cr923ed 16323380

[31] Haahr A Kirkevold M Hall EOC Østergaard K. Living with advanced Parkinson's disease: a constant struggle with unpredictability. J Adv Nurs. (2011) 67:408–17. 10.1111/j.1365-2648.2010.05459.x 20946567

[32] Soundy A Stubbs B Roskell C. The experience of Parkinson's disease: a systematic review and meta-ethnography. Scie World. (2014) 2014:613592–519. 10.1155/2014/613592 25525623 PMC4265687

[33] Levack WMM Weatherall M Hay-Smith EJC Dean SG McPherson K Siegert J. Goal setting and strategies to enhance goal pursuit for adults with acquired disability participating in rehabilitation. Cochrane Database Syst Rev. (2015) 7:CD009727. 10.1002/14651858.CD009727.pub2 26189709 PMC8941379

[34] Seppola-Edvardsen T Andersen RS Risør MB. Sharing or not sharing? balancing uncertainties after cancer in Urban Norway. Health, Risk Soc. (2016) 18:367–84. 10.1080/13698575.2016.1262943

[35] Lloyd A Bannigan K Sugavanam T Freeman J. Experiences of stroke survivors, their families and unpaid carers in goal setting within stroke rehabilitation: a systematic review of qualitative evidence [Review]. JBI Database System Rev Implement Rep. (2018) 16:1418–53. 10.11124/JBISRIR-2017-003499 29894410

